# Updated results and correlative FDG-PET analysis of a phase IB study of vemurafenib and cobimetinib (MEK inhibitor [GDC-0973]), in advanced *BRAF^V600^*- mutated melanoma (BRIM7)

**DOI:** 10.1186/1479-5876-12-S1-O7

**Published:** 2014-05-06

**Authors:** Igor Puzanov, Grant McArthur, Rene Gonzalez, Anna Pavlick, Omid Hamid, Thomas F Gajewski, Ming Yin, Jill Fredrickson, Nicholas Choong, Antoni Ribas

**Affiliations:** 1 Vanderbilt-Ingram Cancer Center, Nashville, TN, USA; 2 Peter MacCallum Cancer Centre, East Melbourne, Victoria, Australia; 3 University of Colorado Comprehensive Cancer Center, Aurora, CO, USA; 4 New York University Medical Center, New York, NY, USA; 5 The Angeles Clinic and Research Institute, Los Angeles, CA, USA; 6 University of Chicago, Chicago, USA; 7 Genentech, South San Francisco, CA, USA; 8 The Jonsson Comprehensive Cancer Center at University of California, Los Angeles, CA, USA

## Background

BRIM7 is a phase 1B study evaluating the safety and efficacy of combined BRAF and MEK inhibition with vemurafenib + cobimetinib. The utility of FDG-PET as an early predictor of clinical benefit was also evaluated in this study.

## Materials and methods

Eligible patients (pts) had advanced *BRAF^V600^*-mutated melanoma and ECOG PS 0-1 and were either naïve to BRAF inhibitor (BRAFi-naïve) or had disease progression on vemurafenib (vem-progressor). Pts in the dose-escalation portion received vemurafenib 720 or 960 mg BID continuously and cobimetinib 60, 80, or 100 mg QD 14 days (d) on/14 d off (14/14); 21 d on/7 d off (21/7); or continuously (28/0). Two dose levels were expanded: vemurafenib (720 mg and 960 mg BID) + cobimetinib 60 mg QD 21/7. FDG-PET scans were performed at baseline and on Day 15 of Cycles 1 and 2. Correlation between tumor glucose metabolism changes, baseline tumor burden and target lesion responses were evaluated.

## Results

128 pts were treated with vemurafenib + cobimetinib as of 21 June 2013; male 60%, median age 55 y (19-88), stage M1c 76% and BRAFi-naive 49%. Median duration of follow-up in vem-progressor and BRAFi-naïve pts was 3 and 10 months, respectively. Most adverse events (AE) were mild to moderate in severity and vem-progressor pts reported fewer AEs compared to BRAFi-naïve pts. Most common AEs in BRAFi-naïve pts (n=63) were non-acneiform rash (89%), diarrhea (81%), photosensitivity/sunburn (70%), fatigue (67%) and liver test abnormality (59%). Most frequent grade ≥3 AEs in BRAFi-naïve pts were liver test abnormality (19%), non-acneiform rash (13%), arthralgia (11%) and fatigue (10%).

BRAFi-naive pts attained 85% confirmed response rate (RR), including 10% complete responses; median PFS was not reached at 10 months follow-up. Vem-progressor pts attained 15% confirmed RR, stable disease of 43%, and median PFS of 2.8 months. Preliminary FDG-PET analysis showed that the magnitude of reduction in tumor glucose uptake correlated with maximal tumor reduction, but the degree of correlation varied across time and in BRAFi-naïve and Vem-progressor pts.

## Conclusions

Vemurafenib + cobimetinib can be safely administered at the respective single-agent MTDs of vemurafenib (960 mg BID) and cobimetinib (60 mg 21/7). Preliminary efficacy of the combination is encouraging in BRAFi-naive patients. FDG-PET is a potentially useful marker of early biologic response to the combination.

